# Geriatric patient in the practice of emergency medical teams – observation in 2020–2022

**DOI:** 10.3389/fmed.2023.1270486

**Published:** 2023-12-20

**Authors:** Łukasz Czyżewski, Łukasz Dudziński, Janusz Wyzgał

**Affiliations:** ^1^Department of Geriatric Nursing, Faculty of Health Sciences, Medical University of Warsaw, Warszaw, Poland; ^2^Emergency Medical Department, John Paul II Academy, Biała Podlaska, Poland; ^3^Department of Nephrologic Nursing, Faculty of Health Sciences, Medical University of Warsaw, Warsaw, Poland

**Keywords:** emergency medical team, geriatrics, 90+, health risks, medical interventions

## Abstract

**Purpose:**

Analysis of interventions of medical rescue teams for geriatric patients in a three-year period, taking into account the causes, circumstances, medical management, pharmacology.

**Materials and methods:**

The study included a 3-year retrospective analysis of the trips of medical rescue teams in the northern part of the Lubelskie Voivodeship in the period from January 1, 2020 to December 31, 2022. The data comes from medical documentation. Interventions caused by a health risk in a patient in the 90+ age group were qualified as events. 897 EMT interventions were qualified (2020–327, 2021–269, 2022–301) constituting 4.29% of all interventions carried out in the operational area. In addition, a quantitative analysis of a comparative group of patients aged 80–89 was performed.

**Results:**

It was shown that the time of rescue activities was the longest for injuries and the shortest for mental disorders (60 ± 31 vs. 43 ± 21 min). It was shown that specialist EMT teams (S) were statistically significantly more often called for cardiology disorders (63%). It was shown that pharmacological agents were used statistically significantly more often in respiratory disorders (83%) compared to neurology disorders (47%, *p* < 0.001). It was also shown that patients whose call was caused by neurology disorders were statistically more often transported to the emergency department (*N* = 76, 76%, *p* < 0.001).

**Conclusion:**

The causes of calls regarding disorders of the circulatory and respiratory systems most often require the implementation of pharmacology during EMT interventions, mainly short-term and symptomatic drugs. Interventions to rural areas dominate in the presented analysis in each year of the analysis and in each group of reasons for calls, which may be associated with more difficult access to a primary care physician. Most EMT interventions concern exacerbation of chronic diseases. Transport to the hospital was necessary mainly due to neurological and traumatic calls.

## Introduction

State Medical Rescue (SMR) system plays an important role in ensuring the safety of Polish residents. The system consists of emergency medical teams (EMT) and hospital emergency departments (ED). The field teams are divided into ground teams (ambulance) and air teams (helicopters) at the Helicopter Emergency Medical Service (HEMS) bases (1, 2).

SMR are responsible for interventions to people in a state of sudden health threat. The person calling medical teams is most often the family of the injured person or a bystander of the incident. Every day, emergency medical services respond to thousands of events that require medical assistance to injured people: emergencies, exacerbation of chronic diseases, injuries, mental disorders (3, 4).

According to data from the Central Statistical Office (CSO) in 2022, 1,592 EMT’s intervened more than 3 million times. SMR based on paramedics, who are the most numerous in the system. Teams with a doctor constitute approximately 20%, but in recent years there has been a downward trend in the number of these teams (5, 6).

According to Eurostat data, in the European Union (EU) countries, there has been a slow increase in the number of older people since 2002, and life expectancy has been systematically increasing, and since 2020 there has been a slight decline in life expectancy (0.3) after taking into account the effects of the COVID-19 pandemic. Life expectancy in the EU in recent years has been as follows:

- 2017 – 80.9 years (almost 7.8 years more for women),
- 2019 – 81.3 years,
- 2020 – 80.4 years,
- 2021 – 80.1 years (approximately 5.7 more for women) (7).

In Poland, women live on average longer than men, with the average life expectancy being almost 8 years longer (74.1 men, 81.8 women) (8, 9).

The diseases most often diagnosed in the elderly include cardiovascular diseases, such as arterial hypertension (HT), ischemic heart diseases, respiratory diseases, bone weakness (osteoporosis), diabetes, vision and hearing disorders, as well as dementia and related cognitive disorders (10).

The aging process of Europe’s population is predicted by demography specialists. The reason for this state of affairs is the low birth rate and the development of medicine, access to new technologies, imaging diagnostics, modern generations of drugs, and specialized therapies (11).

According to the World Health Organization (WHO), old age is divided into young-old age – 60-75 years old, old age – 75-90 years old and longevity – 90+ years old (12).

Neurodegenerative diseases are incurable, however, early diagnosis and initiation of treatment is important, slowing down the development of dementia diseases and mitigating their course. Drawing the attention of the society to the disorders of the elderly may mobilize many families to diagnose their relatives by directing them to early symptoms, which may translate into effective therapy that delays the development of some disorders in accordance with the principle: “prevention is cheaper and better than cure” (13).

Low-energy bone injuries in the course of osteoporotic changes ensuing from prosaic everyday functioning causes (falling as a result of tripping or dizziness) lead to serious health consequences. Bone injury in the elderly person is difficult to heal, and surgical osteosynthesis is often impossible due to comorbidities. Osteoporosis is more common in post-menopausal women. It is a metabolic bone disease characterized by low bone mass and impaired bone microarchitecture. This results in greater bone fragility and susceptibility to fractures (9, 14).

### Purpose

Analysis of interventions of medical rescue teams for geriatric patients (old age) over a three-year period, taking into account the causes, circumstances, medical treatment, and pharmacology. The indirect aim is to observe quantitative data (number of patients) during medical interventions for the oldest part of the region’s population.

## Materials and methods

### Research design

The research included a 3-year retrospective analysis of dispatches by EMTs from the northern part of the Lubelskie Province (eastern border of the European Union). The analysis covers the period from 01.01.2020 to 31.12.2022. The data was obtained from the official documentation of the dispatch units of the SMR system:

- dispatch order card (DOC) – this part is prepared by the medical dispatcher (MD) at the Rescue Notification Center (RNC),
- Medical Rescue Operations Card (MROC) – completed by the head (leader) of the EMT, i.e.:
- doctor – specialist team (S),
- a paramedic or nurse of the system - basic team (B) (3).
- In the operational area covered by the observation there are four round the clock teams, 2 type B and 2 type S teams. In addition, in the operational area there is a hospital ED in the county hospital, where the field teams transfer the transported patients for further hospital treatment, observation and diagnostics.

### Research setting

Interventions that met the inclusion criteria were analyzed, taking into account the date and time of the intervention, the length of the intervention expressed in minutes, the location of the event (urban and rural areas), the type of EMT, the age and gender of the patient, rescue procedures, the use of pharmacological agents, medical diagnosis (as per ICD – 10). An analysis of the causes of calls was made, which were grouped according to the following classification: mental disorders, cardiology disorders, neurology disorders, injury, respiratory disorders, metabolism/nutrition, infection, other (pain/allergy/cancer).

### Ethical considerations

The consent of the Director of the unit carrying out dispatch orders in the examined operational area for access to medical records was obtained in June 2022. All personal data (of patients, medical staff, and cooperating services) remained anonymous and were not used for analysis purposes. The analysis was carried out in accordance with the Declaration of Helsinki. Accordingly, the local bioethics committee was not asked for opinion and consent to carry out the research.

### Statistical analysis

Results concerning quantitative variables were presented as average values ± standard deviation. In the comparative characteristics of characteristic of EMT interventions according to year of intervention and reasons of the call, a one-way analysis of variance (ANOVA). Simple linear regression analysis (Pearson) was applied to detect and describe the strength and direction of correlations of time of intervention to age of patients. Qualitative variables (age, sex) were presented as quantity (n) and percentage values of the whole group (%), while proportions in groups were assessed with a Chi-squared test. Statistica 13 software (StatSoft Inc., Tulsa, OK) was used in the statistical analysis. *p* < 0.05 was adopted as the significance level.

### Inclusion criteria

1. EMT calls to a patient aged 90+.
2. dates of commencement of EMT interventions between 1.01.2020 at 0.00 a.m. and 31.12.2022 at 11.59 p.m.
3. the intervention for patients aged 80–89 was included as a comparison group (quantitative correlation – epidemiological data).

Justification for selecting the inclusion group in the study.

It was decided to select patients aged 90+ because, according to statistics, this age is above the average life expectancy for the inhabitants of Poland.

Moreover, according to the Polish insurance system, the estimated life expectancy is calculated for the purposes of granting and the hypothetical amount of capital pensions and annuities. The tool for these estimates are Pension Tables calculated according to the following scheme:

YM – current age (Y – years, M – months) + X(m) – estimated age of survival expressed in months.

The maximum age included in the Tables is 90 years for both sexes and estimates of further life expectancy are calculated from this age in Table 1 (15).

**Table 1:** Life expectancy tables for age 90 in Poland.

	Age 90: months											
	I	II	III	IV	V	VI	VII	VIII	IX	X	XI	XII
Life expectancy (months)	49,3	49,1	48,8	48,5	48,2	48,0	47,7	47,4	47,2	46,9	46,6	46,4

Source: World Health Organization, (15).

### Exclusion criteria

1. absence of the patient at the place of call – incorrect address details.
2. false calls to the emergency phone number,
3. absence of the patient at the place of call, while waiting for the EMT, the family transported the patient to the hospital or general practitioner on their own.

## Results

Using the inclusion and exclusion criteria, 897 EMT dispatches were selected from the analysis, accounting for 4.29% of all interventions (Table 2). It is worth emphasizing that interventions were analyzed, not patients. In the analysis, there is a difference between the number of interventions and the patient population, which results from repeated calls to the same patients (a maximum of 11 interventions to the same patient in the period covered by the analysis), the results are presented in Table 3.

**Table 2:** General characteristic of all interventions.

Year	Overall events	N included in the analysis	%
2020	7,054	327	4,63
2021	7,069	269	3,80
2022	6,769	301	4,44
Total	20,892	897	4,29

**Table 3:** Patients included in the analysis for whom MRT intervened more than once (data for *n* > 3).

Interventions	Gender/ age *	Interventions	Gender/ age *
10	W102	4	M92
7	W92	3	W92
5	W100	3	W91
5	W91	3	W94
4	W93	3	W91
4	W92	3	M96
4	M90	3	W94

W, woman; M, man. *Age of the patient at the time of the last intervention included in the analysis.

In total, 168 re-interventions to 93 patients were performed in the 3-year analysis. Apart from the data in Table 3 describing 14 patients, up to 28 patients were intervened upon 3 times, and up to 51 patients twice. Repeated interventions accounted for 18.72% of all calls in this age group (90+), and they concerned women *n* = 142 (84.52%), and men *n* = 26 (15.48%).

Analysis of Simple linear regression analysis (Pearson) between time of intervention and age of patients showed no statistical significance (*R* = −0,049; *p* = 0.140).

Most interventions for the 90+ age group occurred in rural areas (76%). The results indicate the vast majority of interventions addressed to women (75.80%). Deaths concerned 5% (*N* = 46) of the study group of patients. Over the years of the analysis (2020 vs. 2021 vs. 2022), the following did not change: the location of the intervention (*p* = 125); intervention time (*p* = 765); call frequency by gender (*p* = 0.680); share of “S” teams (*p* = 0.151); frequency of pharmacotherapy use (*p* = 0.470) and number of deaths (*p* = 0.389). There was only a statistically significant difference in the frequency of patient transport to the ED (*p* = 0.034) and the age of the patients (*p* = 0.017; Table 4).

**Table 4:** Univariate comparison of characteristic of EMTs interventions according to year of intervention.

	2020	2021	2022	All	*p*
N	327	269	301	897	
Age, y	93 ± 3	93 ± 3	93 ± 2	93 ± 2	0.017
Time, min	52 ± 26	52 ± 23	53 ± 24	52 ± 24	0.765
Sex, male, n(%)	74 (23)	69 (26)	74 (25)	217 (24)	0.680
S EMT, n (%)	175 (54)	152 (57)	146 (49)	473 (53)	0.151
City, n(%)	66 (20)	65 (24)	85 (28)	216 (24)	0.125
Pharmacology, n(%)	209 (64)	167 (62)	202 (67)	578 (65)	0.470
Death, n(%)	12 (4)	20 (7)	14 (5)	46 (5)	0.389
Transfer to ED, n(%)	190 (58)	140 (52)	189 (63)	519 (58)	0.034

ED, emergency department; S EMT, specialist Emergency Medical Team.

**Figure 1:**
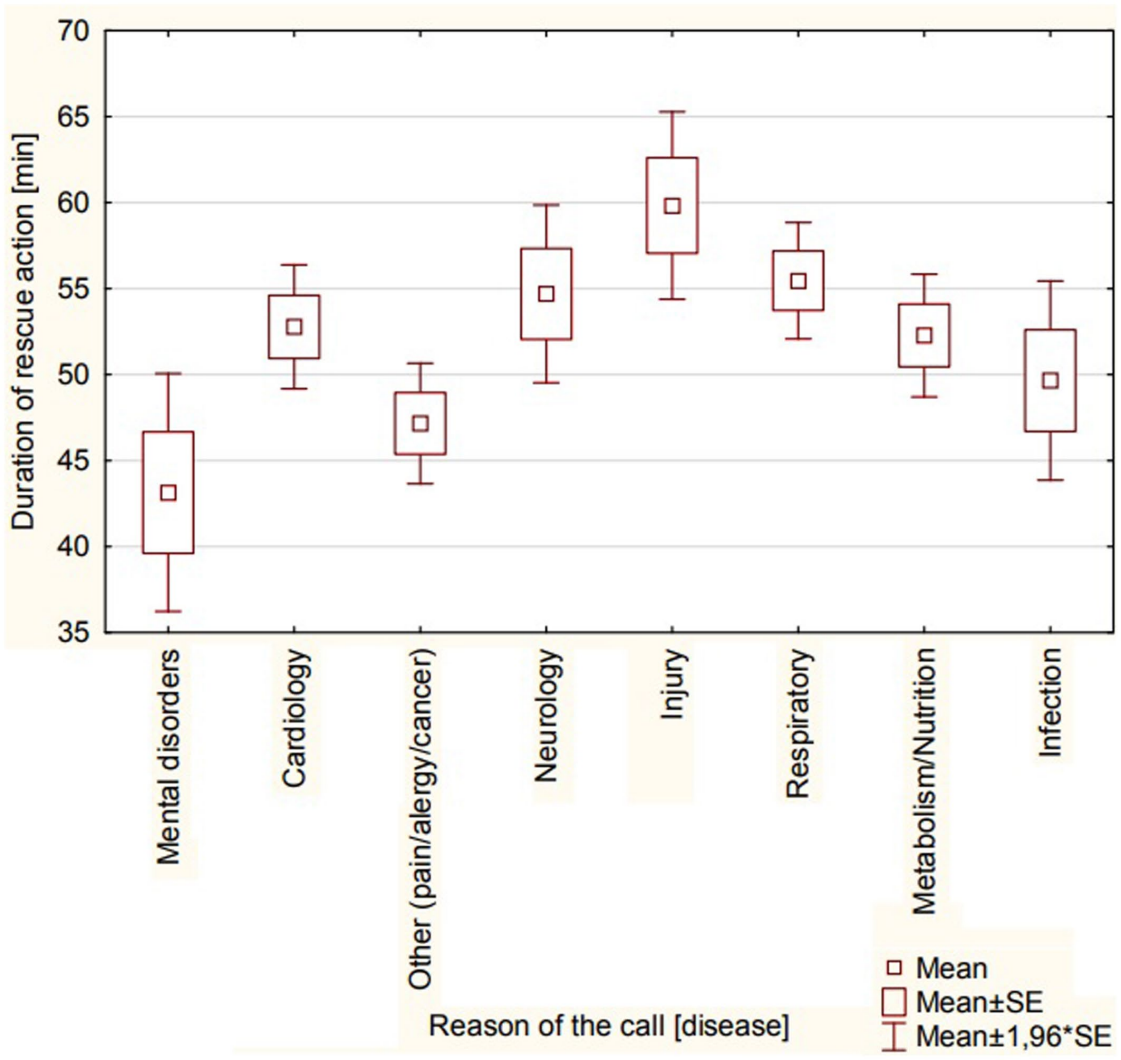
Duration of rescue action [min] according to reason of the call [disease].

An analysis of the characteristics of the intervention and the reasons for the calls was made. Statistically significant differences were found in the duration of rescue action [min] depending on the reason for the call (*p* < 0.001; Figure 1). It was shown that the time of rescue actions was the longest for injuries and the shortest for mental disorders (60 ± 31 vs. 43 ± 21 min). It was shown that specialist EMT teams (S) were statistically significantly more often called to cardiology disorders (*N* = 117, 63%). It was shown that pharmacological agents were used statistically significantly more often in respiratory disorders (*N* = 119, 83%) compared to neurology disorders (*N* = 47, 47%, *p* < 0.001). It was also shown that patients whose call was caused by neurology disorders were statistically more often transported to the ED (*N* = 76, 76%, *p* < 0.001). In the comparative analysis of the year (2020 vs. 2021 vs. 2022) according to the causes of calls, it was shown that the share of cardiology disorders decreased in the analyzed years (41% vs. 32% vs. 27%, *p* = 0.003). There was no statistically significant effect of the affected person’s age (*p* = 0.319) and the location of the incident (*p* = 194; urban vs. rural) on the indicated reasons for the frequency of calls (data in Table 5).

**Table 5:** Univariate comparison of characteristic of MRTs interventions according to reasons of the call.

Parameters	Mental	Cardiology	Neurology	Injury	Respiratory	Metabol./Nutr.	Infection	Other	*p*
N	35	187	100	124	144	128	26	112	
Age, y	94 ± 4	93 ± 3	93 ± 3	93 ± 2	93 ± 2	93 ± 2	93 ± 3	93 ± 3	0,319
Duration, min	43 ± 21	53 ± 25	55 ± 26	60 ± 31	55 ± 21	52 ± 21	50 ± 15	47 ± 19	<0,001
Sex, male, n(%)	9 (26)	34 (18)	17 (17)	36 (29)	40 (28)	26 (20)	10 (39)	34 (30)	0,031
S EMTs, n(%)	18 (51)	117 (63)	58 (58)	48 (39)	79 (55)	64 (50)	15 (58)	48 (42)	0,002
Localization, city, n(%)	13 (37)	51 (27)	26 (36)	30 (24)	24 (17)	29 (23)	7 (27)	23 (21)	0,194
Pharmacology, n(%)	16 (46)	137 (73)	47 (47)	74 (60)	119 (83)	95 (74)	22 (85)	68 (61)	<0,001
Transfer to ED, n(%)	11 (31)	109 (58)	76 (76)	89 (72)	88 (61)	80 (63)	13 (50)	53 (47)	<0,001
Year, n(%)									0,003
	2020	13 (37)	76 (41)	39 (39)	45 (36)	39 (27)	57 (45)	8 (31)	38 (34)
	2021	14 (40)	61 (32)	23 (23)	31 (25)	59 (41)	33 (26)	9 (35)	23 (21)
	2022	8 (23)	50 (27)	38 (38)	48 (39)	46 (32)	38 (30)	9 (35)	51 (46)

ED, emergency department; S EMT, specialist Emergency Medical Team.

**Figure 2:**
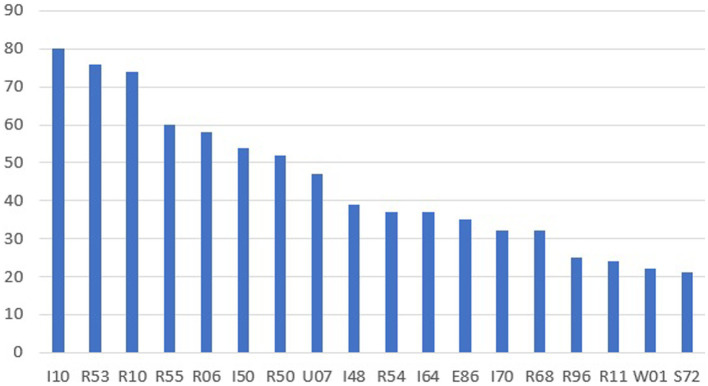
ICD-10 diagnoses for n > 20. I10-hypertension, R53-bad mod and fatigue, R10-stomachache, R55-fainting and collapse, R06-breathing disorders, I50-heart failure, U07-multisystem inflammatory syndrome associated with COVID-19, I48-atrium fibrillation, R54-old age, E86-excessive fluid loss, I70-atherosclerosis, R68-other general symptoms and signs, R96-other sudden death from an unknown cause, R11-nausea and vomiting, W01-falling on the same level due to tripping, slipping, S72-fracture of the femur.

Medical diagnoses described in EMT intervention charts were analyzed according to the International Statistical Classification of Diseases and Related Health Problems (ICD-10) guidelines (9). Figure 2 presents the most frequently used ICD-10 medical diagnoses in the analysis. 1,248 ICD-10 diagnoses were used for 897 interventions. In 351 interventions, 2 diagnoses were entered, mainly in patients with trauma (essential diagnosis and ICD-10 code describing the circumstances or mechanism of the injury).

The analyzed age group (90+) was compared with the age group of 80–89 years – the total number of interventions broken down by patients’ gender was included in Table 6.

**Table 6:** Comparative group – interventions for patients aged 80–90 years.

2020		2021		2022	
W	M	W	M	W	M
678	355	718	332	711	334

M, interventions to men; W, interventions to women.

The purpose of the comparison was to show the scale of the number of interventions for patients in the age groups 89–89 vs. 90+. In each analyzed year, the population aged 80–89 was significantly more numerous than the main analyzed population (90+), additionally, it was found that in the comparison group, similarly to the study group, interventions for women had prevailed. In total, in the analyzed period, there were 3,128 interventions for patients aged 80–89 (2,107 for women, 1,021 for men) – Table 5. In total, for the age groups 80–89 and 90+ EMTs intervened a total of 4,025 times, which is 19.26% of all completed interventions in the three-year period.

**Figure 3:**
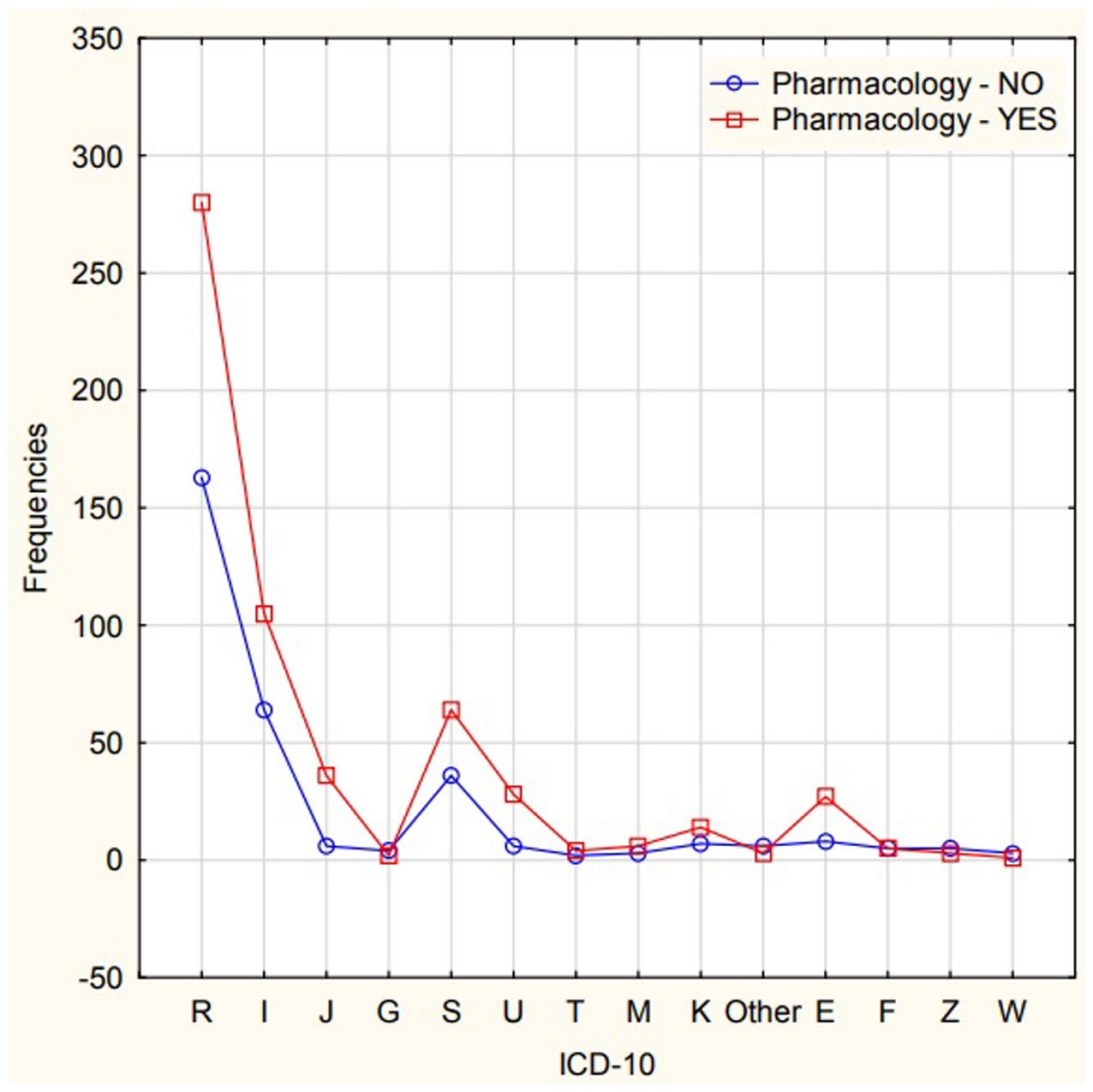
Interactions of frequency between the use of pharmacotherapy and ICD-10 groups.

Pharmacological treatment was used in 597 interventions (patients were administered from 1 to 4 preparations, including medical oxygen). The most common were drip infusions of 250 and 500 mL – 169 interventions, dexamethasone 121 times, metamizole 161 times, medical oxygen 89 times, ketoprofen 44 times, metoclopramide 37 times. According to Figure 3, pharmacotherapy was mainly used for ICD-10 diagnoses from group R (the largest group), group I (hypertension, heart failure, irregular heartbeat, stroke), and group S (this group includes injuries, mainly S72 – fracture of the femoral neck).

## Discussion

Polish population is aging, as is most of the developed countries of the EU, of which Poland forms the eastern border. Trends in life expectancy rates in Europe show a shift in the limits of life expectancy, with an indication of longer life for women (16, 17). The results of our analysis are in line with these trends. In the oldest part of the population to which EMTs intervened in the three-year analysis, i.e., 80–89 years and 90+, there were definitely more female patients.

Geriatric patients are often unable to function independently, being cared for by their families or professional nursing homes. In our own analysis, there were cases of intervention of a medical team to a nursing home (senior home), the location of the event was not the main purpose of the analysis. A 24-h nursing home cannot replace a hospital. Accordingly, a significant part of the interventions required transporting patients to hospital treatment, e.g., exacerbation of a chronic disease, COVID-19 infection, bone injury with fracture in the course of osteoporosis in the low-energy mechanism (e.g., tripping and falling from own height, falling out of bed, out of a chair). Our own observations confirm the results of Głuszko et al. (9), who described the health problems of the elderly associated with bone decalcification and a high risk of fractures. In our own study, the diagnosis S72 (femoral neck fracture) predominated among the injuries.

Interesting data are presented by Linder et al. (18) on a geriatric patient in the Hospital ED. The results indicate that the geriatric patient accounts for 12–24% of all hospitalized patients, and the trends indicate that this group will increase in the future. The data obtained in our own observation indicate that 58% of patients need to be transported to the ED. Interventions of medical teams often cannot end at the call point, they require the patient to be transported to the hospital for further diagnosis, consultation and observation.

The period of our analysis is partly connected with the period of the COVID-19 pandemic. Interventions for patients with suspected COVID-19 (*n* = 47) accounted for 5.2% of the total. These were U07 diagnoses. Medical teams are equipped with rapid tests (nasopharyngeal swabs) that give results in a short time. However, full confirmation is provided by the polymerase chain reaction (PCR) laboratory test, which is available at the hospital. Noteworthy are the diagnoses R06 (*n* = 58) and R50 (*n* = 52), which could potentially be the beginning of COVID-19 infection, but a quick test at the pre-hospital stage did not confirm the infection (19–22).

The total number of SMR dispatches in the observed operational area did not change significantly during the COVID-19 period and the years preceding it. Data with the total number of interventions since 2015 come from published observations of the functioning of EMTs and interventions to other groups of patients. In 2015, there were 5,782 interventions, 2016–7,830, 2017–7,890, 2018–7,580, 2019–7,624, 2020–7,054, 2021–7,069 and 2022–6,769 (Mean 7,199,7, SD 700,2) (23, 24).

Most interventions in our observation occurred in rural areas (*n* = 681), which may be related to more difficult access to a general practitioner (medical centers are more often located in cities). In addition, in rural areas, elderly people often live alone, and their families (professionally active) choose to live in the city for employment reasons. In the authors’ opinion, another factor of frequent EMT interventions in rural areas may be insufficient care provided by the family. Hard work in agriculture and lack of time for proper care, lack of funds to finance a nursing home, inability to transport seniors for check-ups at the general practitioner’s office, result in exacerbation of chronic diseases and the need to call a EMT.

Pharmacotherapy in interventions up to the age group of 90+ consists mainly of symptomatic drugs. Błeszyńska-Marunowska et al. draws attention to the difficulties of pharmacotherapy of seniors due to changes in metabolism, multi-morbidity and great interest in over-the-counter drugs, leading to polypharmacy of seniors, which consequently requires the implementation of combined medical and pharmaceutical care for geriatric patients. Our own observations did not include the analysis of self-pharmacotherapy by patients, only drugs administered by EMTs, mainly symptomatic ones, oxygen and drip infusions (25).

In the case of pharmacotherapy used by EMTs, it is important to limit the regulations (Minister of Health) regarding the number of pharmacological agents that EMTs have at their disposal, and the differences in the use of pharmacology between S teams (greater number of pharmacological agents due to the greater scope of physicians’ authority) and B teams (without a physician) (26–28).

Nadolny et al. analyzed MRT interventions in Poland during the COVID-19 pandemic, he found that the overall number of interventions had decreased compared to the previous period. The authors classified all EMT interventions categorized by the reason for the call, including: death, syncope, mental disorder, traffic accident, trauma, stroke, sudden cardiac arrest, shortness of breath, pregnancy, chest pain, circulatory disorders. Our research also classified grouped interventions matched to the age group of patients (29).

Celiński et al. also analyzed geriatric patients in the EMT practice in eastern Poland. The results from 2019 to 2020 confirm our observations. Two thirds of the patients were transferred to the ED, mainly due to cardiovascular diseases, injuries arising from external causes and respiratory diseases (30).

Death in this age group is not very common. Diagnoses R96 and R98 (*n* = 19 – items not included in Figure 1 results for *n* > 20) occurred in a total of 46 interventions. Some of the patients in very serious condition were transported by medical teams to the ED. The consent to obtain the data did not cover the hospital documentation. Therefore, the authors do not know how many patients died in the subsequent hours of hospital stay. Based on our own data on repeated interventions (*n* = 168) and data from the CSO, it can be assumed that pre-hospital rescue, equipment, experience of medical teams, procedures, and pharmacotherapy have a positive impact on the life expectancy of the population (31).

Interestingly, one intervention in the “rural area” group ended with the transfer of the patient to the HEMS team, due to the patient’s serious condition and the long distance to the hospital. This intervention shows that pre-hospital rescue in Poland is at a high level, has efficient coordination of various types of medical teams (ground and air), using all possible forces and means to save lives, regardless of the patient’s age.

### Limitations

The interventions included in the analysis in accordance with the assumed purpose of the paper account for a clear percentage of all EMT interventions, constituting a challenge for units providing pre-hospital rescue. The figures for interventions for groups 80–89 and 90 show that the aging population often needs pre-hospital assistance. The obtained consent for access to data covered only the documentation of the EMT dispatch teams, no access was granted to the documentation of further hospital treatment.

## Conclusion

The causes of calls for disorders of the circulatory and respiratory systems most often require the implementation of pharmacology during EMT interventions, mainly short-term and symptomatic drugs. Interventions to rural areas dominate in the presented analysis in each year and in each group of reasons for calls, which may be associated with more difficult access to a primary care physician. Transport to the hospital was necessary mainly for neurological and traumatic calls, and the intervention time was the longest in the group of trauma patients, which is related to bone injuries and the need to transport to a facility further than the local hospital. The tendency of repeated interventions to the same patients may confirm the unstable health of this age group, the lack of proper care, the effectiveness of previous EMT interventions. Deaths in this age group are not significantly common during EMT interventions, which may be related to patients’ stay in hospital wards and care facilities in the last period of life.

## Data availability statement

The original contributions presented in the study are included in the article/supplementary material, further inquiries can be directed to the corresponding author.

## Author contributions

ŁC: Writing – review & editing, Conceptualization, Data curation, Formal analysis, Software, Supervision. ŁD: Data curation, Supervision, Writing – review & editing, Investigation, Methodology, Visualization, Writing – original draft, Resources. JW: Formal analysis, Resources, Supervision, Validation, Visualization, Writing – review & editing.
